# Endothelial dysfunction in single ventricle physiology and the Fontan circulation – What lies ahead

**DOI:** 10.1016/j.ijcchd.2025.100589

**Published:** 2025-04-28

**Authors:** Raksheeth Agarwal, Louise E. Coats, Ali N. Zaidi

**Affiliations:** a Department of Medicine, Jacobi Medical Center/ Albert Einstein College of Medicine, Bronx, NY, USA; b Adult Congenital Heart Unit, Freeman Hospital, Newcastle Upon Tyne Hospitals NHS Foundation Trust, Newcastle Upon Tyne, UK; c Population Health Sciences Institute, Newcastle University, Newcastle Upon Tyne, UK; d Mount Sinai Adult Congenital Heart Disease Center, Mount Sinai Heart, New York, NY, USA

**Keywords:** Single ventricle congenital heart disease, Endothelial dysfunction, Glenn circulation, Fontan circulation

## Abstract

Endothelial dysfunction is characterized by a vasoconstricted, pro-coagulative, and pro-inflammatory phenotype and is known to play a role in several chronic non-communicable diseases. Several biophysical and biochemical markers have been developed to assess endothelial function clinically. Its relevance in individuals born with single-ventricle congenital heart disease (SV-CHD) is increasingly recognized. Endothelial dysfunction has been observed in all stages of palliation in SV-CHD patients. Several mechanisms possibly contribute, including genetic factors, hypoxia, loss of pulsatility of blood flow, neurohormonal and sympathetic overactivation, and oxidative stress. Clinically, it possibly contributes to impaired pulmonary flow, exercise limitation, thromboembolisms, liver dysfunction, and adverse pregnancy outcomes. Based on this information, several therapeutic targets have been proposed such as early surgical and exercise interventions, pulmonary vasodilators, and other common pharmacological agents. However, much remains unknown and future studies must unravel the relationship of endothelial dysfunction with this complex patient group, ultimately improving their clinical care.

## Highlights

• Endothelial dysfunction is present at each stage of palliation in SV-CHD.
• Mechanisms include hypoxia, loss of pulsatility, and oxidative stress.
• It influences key outcomes such as pulmonary hemodynamics and exercise tolerance.
• Trials have failed to identify medications that can improve endothelial function.
• Nevertheless, there is a strong rationale for it as a potential therapeutic target.

## 1 Introduction

The most complex congenital heart defects involve a single functional ventricle, such as tricuspid atresia, double inlet left ventricle, and hypoplastic left heart syndrome. Roughly 1 in 10,000 individuals have single ventricle congenital heart disease (SV-CHD), and the prevalence among adults is continuing to grow. Better survival is a consequence of the success of the staged Fontan procedure. The initial stage is typically the bidirectional Glenn (BDG) procedure, where the ligated superior vena cava (SVC) is connected to the confluent pulmonary arteries [1]. Completion of the Fontan circulation is achieved through redirection of inferior vena cava (IVC) blood to the pulmonary arteries. Initially this was achieved with the atrio-pulmonary connection (APC), which was modified with the lateral tunnel (LT) and eventually the extra-cardiac total cavo-pulmonary connection (TCPC) [2]. In the Fontan circulation, blood flows directly to the lungs from the systemic veins without the presence of a sub-pulmonary ventricle whilst the single functional ventricle directly supports the systemic circulation [3]. The result is the formation of a neo-portal system, with systemic and pulmonary capillary beds connected in series without an intercedent ventricular pump [4].

Whilst the Fontan circulation has dramatically improved survival for SV-CHD, it creates a unique physiology that is complicated by cardiac dysfunction, thromboembolism, liver and kidney disease, relative pulmonary hypertension, and protein losing enteropathy [2,5]. Fontan patients also exhibit poor exercise tolerance [5], a parameter closely related to mortality in CHD populations [6]. Multi-organ failure is insidious in onset and is thought to occur due to high resistance across the pulmonary neo-portal system, causing systemic venous congestion and limiting cardiac output. Cardiac output, typically regulated by the heart, is instead managed by blood flow through this passive neo-portal system [4]. On a mechanistic level, endothelial dysfunction is believed to play a key role in the development of complications in single ventricle congenital heart disease (SV-CHD) and may influence prognosis. This review seeks to summarize the current evidence of endothelial dysfunction at each stage of SV-CHD palliation, assess its impact, and highlight key questions for future research.

## 2 Endothelial function and its assessment

### 2.1 The clinical significance of endothelial function and dysfunction

**Fig. 1:**
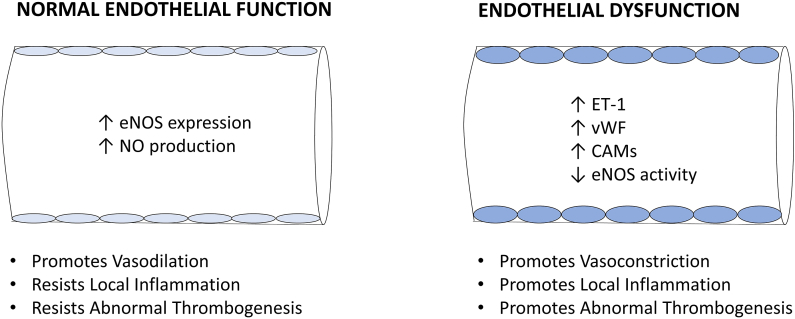
Normal endothelial function (left) and endothelial dysfunction (right).

The endothelium (Fig. 1) is a single layer of squamous endothelial cells that line the luminal side of the vascular system. Vascular tone is governed by a balance between endothelial vasodilators (nitric oxide [NO], prostacyclin, and hyperpolarizing factors) and vasoconstrictors (endothelin-1 [ET-1], angiotensin-II, and superoxide). Endothelial nitric oxide synthetase (eNOS) derived NO also prevents abnormal cell adhesion and smooth muscle proliferation. Thus, the endothelium maintains vascular integrity whilst preventing excessive platelet and coagulative activity. Finally, the endothelium also regulates inflammation by controlling the expression of cell adhesion molecules and cytokines [7,8]. Endothelial dysfunction is characterized by a failure of these functions, presenting with a vasoconstricted, pro-coagulative, and pro-inflammatory phenotype [7,8]. Endothelial function declines with age and body mass index (BMI) in both sexes [[9], [10], [11]]. Women are somewhat protected until the age of 70, possibly due to the vasoprotective properties of estrogen [9,10]. Endothelial dysfunction is also linked to chronic inflammation, oxidative stress, and cardiovascular risk factors like hypertension, diabetes, smoking, and hyperlipidemia [7,8]. It plays a role in the progression of diseases such as heart failure, chronic kidney disease, and atherosclerosis [7,8].

### 2.2 Biophysical markers of endothelial function

Vascular reactivity to physical stimuli reflects endothelial function, which forms the basis of its assessment by flow mediated dilation (FMD) and endothelial pulse amplitude tonometry (EndoPAT). In FMD, a manometer cuff is used to occlude and subsequently reperfuse an artery. The resultant increase in shear stress on the vessel wall triggers endothelium dependent vasodilation, which can be quantified using ultrasound. Similarly, in EndoPAT, the change in fingertip blood flow after manometer cuff inflation-deflation is measured [12]. The PAT ratio can be derived from this hyperemic response, which reflects endothelial function [13]. Aside from these physical stimuli, the vasodilatory response to various pharmacological substances (E.g. acetylcholine [ACh]) can be assessed [12].

### 2.3 Biochemical markers of endothelial function

Abnormal expression and activity of eNOS and reduced NO bioavailability reflect endothelial dysfunction [14]. Endothelium derived ET-1, von-Willebrand factor (vWF), and cellular adhesion molecules (e.g. selectins, vascular cell adhesion molecule 1 [VCAM-1], and intercellular adhesion molecule 1 [ICAM-1]) promote vasoconstriction, platelet activity, and inflammation, respectively. Elevated circulating levels of these markers reflect endothelial dysfunction and have been associated with increased cardiovascular risk [15,16].

## 3 Evidence of endothelial dysfunction in the SV-CHD population

### 3.1 Endothelial dysfunction in the pre-glenn single ventricle circulation

Endothelial dysfunction in SV-CHD patients prior to the Glenn procedure is poorly documented. A single study found reduced brachial artery FMD and increased plasma ET-1 in a mixed group of pre- and post-Glenn SV-CHD patients compared to biventricular CHD controls. FMD did not differ according to stage of palliation (pre vs post Glenn), use of angiotensin converting enzyme (ACE) inhibitors, or a previous arch reconstruction. The influence of the primary CHD diagnosis was not explored [17]. Although small, this study suggests that endothelial dysfunction may exist from an early age and potentially pre-date surgical intervention. Further research is needed to confirm these findings and explore the impact of the primary CHD phenotype, as well as potential genetic factors. Such insights could help identify early therapeutic targets that may influence the long-term health trajectory of these patients.

### 3.2 Endothelial dysfunction in the post-glenn single ventricle circulation

In animal models of the classic Glenn procedure (where the SVC is connected unilaterally to the right pulmonary artery), eNOS expression in the right lung is reduced [18,19]. In human BDG patients, pulmonary artery (PA) ACh responsiveness is reduced but nitroglycerine responsiveness is preserved, suggesting impaired endothelial function but normal smooth muscle function [20]. Elevated plasma vWF and reduced thrombomodulin (TM) levels have also been observed [21]. Abnormally low TM levels, possibly due to reduced endothelial expression, are associated with increased cardiovascular risk [22]. Polymorphisms in the TM gene reduce its expression in endothelial cells, increasing the risk of coronary artery disease and thrombosis [23]. In Eisenmenger syndrome, low TM levels are hypothesized to be due to reduced expression in response to chronic hypoxia [24]. Reduced TM levels in Glenn patients may also reflect reduced expression, but arterial saturations have not been found to correlate in this group [21]. It remains to be determined whether the chronically reduced TM levels in SV-CHD patients are due to constitutional reduction in endothelial expression, and if so, what the mechanisms and clinical implications are.

### 3.3 Endothelial dysfunction in Fontan-palliated single ventricle patients

A recent meta-analysis demonstrated that compared to controls, single ventricle and Fontan patients have impaired FMD, reactive hyperemia index (RHI), arterial distensibility, and higher arterial stiffness measured by pulse wave velocity, stiffness index, and augmentation index [25]. Besides this, Fontan-palliated patients also show derangements in biochemical markers of endothelial function, including elevated levels of ET-1[[26], [27], [28], [29]], vWF [28,30], VCAM-1 [28], and angiopoietin-2 [31]. As seen in Glenn patients, TM levels continue to be reduced after Fontan completion [32,33].

There is also evidence of pulmonary vascular dysfunction in Fontan-palliated patients. Their pulmonary arteries have reduced distensibility and a lack of variation in wall shear stress and pulmonary flow across the cardiac cycle, indicating reduced pulsatility [34]. They have an elevated pulmonary vascular resistance (PVR) with significant reduction after NO inhalation [35]. Another study showed an impaired flow response to ACh infusion in the pulmonary arteries [36].

The relationship between oxygen saturation (SaO_2_) and endothelial dysfunction appears complex. While one study reported no correlation between SaO_2_ and pulmonary ACh reactivity, another reported an inverse correlation with soluble P-Selectin [36,37]. Interestingly, a significant correlation between duration of hypoxemia before Fontan completion and reduced brachial artery FMD has been observed [38]. The degree of endothelial dysfunction has not been shown to be affected by the type of initial palliation [36], previous aortic arch surgery [39], systemic ventricular morphology [26,[38], [39], [40]], or current medication (warfarin [33], aspirin [28], and ACE-inhibitors [[38], [39], [40]]). Thrombomodulin levels have been reported to be lower in the APC compared to the TCPC with levels falling as the Fontan duration increases [32]. However other studies have not corroborated this [36,38].

In contrast to the general population, endothelial function does not vary with age, sex, and BMI in young Fontan patients [38,40]. This loss of normal variation is also seen in type 1 diabetics, another young population with endothelial dysfunction [41].

## 4 Possible mechanisms of endothelial dysfunction in the SV-CHD population

**Fig. 2:**
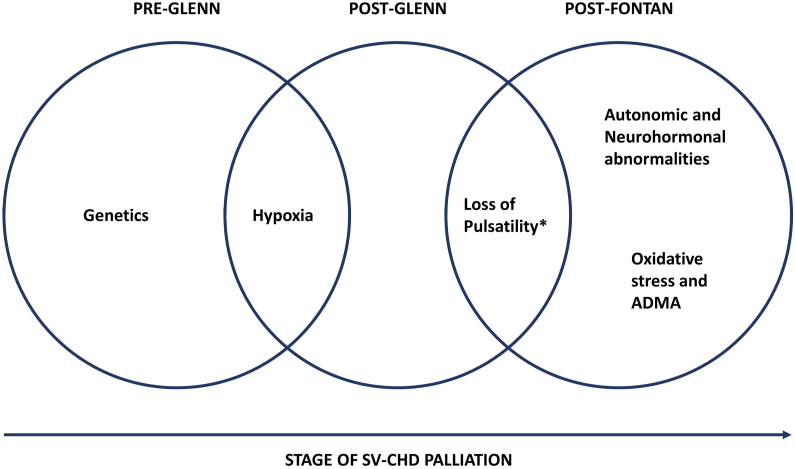
Possible mechanisms of endothelial dysfunction according to stage of SV-CHD palliation. ∗Loss of pulsatility in the pulmonary circuit. ADMA = Asymmetric Dimethyl Arginine.

Endothelial dysfunction is observed at every stage of SV-CHD palliation, and different mechanisms may be hypothesized to contribute at each stage. These are summarized in the figure below (Fig. 2) and discussed in the following sub-sections.

### 4.1 Hypoxia

Infants born with SV-CHDs are cyanotic at birth. A hypoxic environment promotes thrombosis and inflammation by upregulating endothelial expression of tissue factor, plasminogen activator inhibitor-1, cytokines, and cell adhesion molecules, while suppressing TM expression. It also upregulates ET-1 and stimulates the exocytosis of Wiebel-Palade bodies, increasing the levels of vWF and P-Selectin [42]. Reduced expression of eNOS and blunted response to ACh has been noted in infants and adults with cyanotic CHDs [43,44].

Longer duration of exposure to cyanosis before Fontan is correlated with increased endothelial dysfunction after Fontan completion [38], suggesting that early exposure to hypoxia influences long-term endothelial function in SV-CHD patients. This may partially explain why Fontan completion at an older age is associated with poor long-term outcomes [45].

Desaturation and chronic hypoxia persist after the Glenn stage and may continue to influence endothelial function. While arterial saturations improves greatly following Fontan completion, variable degrees of desaturation persists due to fenestrations and collateral development [46]. Evidence on the correlation between desaturation and endothelial dysfunction after Fontan completion is limited and conflicting [36,37]. Hence, hypoxia appear to be more relevant before Fontan completion, but longitudinal cohort studies are needed to explore its effects at all stages of palliation.

### 4.2 Loss of pulsatile flow

Normal endothelial function depends on exposure to shear stress, and it is further enhanced when blood flow is pulsatile. In cultured endothelial cells, exposure to shear stress increases eNOS expression in a dose dependent manner and downregulates ET-1. Nitric oxide production is further increased when these cells are exposed to pulsatile flow [47]. In patients undergoing pulsatile flow cardiopulmonary bypass (CPB), blood concentrations of NO_2_^−^ are higher compared to continuous flow CPB, with no difference in erythrocyte NOS activity, suggesting an increase in endothelial NOS activity [48].

Following BDG, pulsatility in pulmonary arteries is lost as they are supplied passively by the SVC. However, in a subset of patients, some antegrade pulsatile blood flow is preserved due to persistence of systemic-to-pulmonary shunts or a patent right ventricle outflow tract [49]. In the classic Glenn pig model, PA eNOS expression was preserved in the micro-pulsatile group compared to the non-pulsatile group [18]. In human BDG subjects, a strong positive correlation between vascular responsiveness to ACh and pulsatility of the pulmonary arteries was observed [20]. Clinically, an association between preserved antegrade pulsatile blood flow and improved long-term outcomes has been reported. This was speculated to be due to improved PA growth and endothelial function, but not overtly proven [50]. Pulsatility of pulmonary blood flow continues to be reduced after Fontan completion [34,51], and hence this mechanism continues to be relevant at this stage as well.

### 4.3 Autonomic and neurohormonal abnormalities

Fontan-palliated patients experience sympathetic and neurohormonal overactivation, clinically presenting with reduced venous capacitance and increased peripheral vascular resistance. This is thought to be compensatory to maintain perfusion in the setting of a fixed and limited cardiac output [[52], [53], [54]]. An elevation in resting muscle sympathetic nerve activity (MSNA) is observed in Fontan patients [52,53], which has been associated with endothelial dysfunction in healthy and obese individuals [55,56]. Neurohormonal factors including angiotensin-II and ET-1 are also elevated [26,29], which are known to mediate endothelial dysfunction [15,57]. While MSNA is elevated even in Fontan patients with preserved ventricular function [53], ET-1 appears to be higher in those with poorer ventricular function and New York Heart Association (NYHA) functional class [26,52]. Therefore, this mechanism possibly contributes further in a subset of patients functioning poorly. Although ET-1 is elevated in pre-Fontan SV-CHD patients [17], information on other indices of neurohormonal and sympathetic activity in this group is limited.

### 4.4 Asymmetric dimethyl arginine and oxidative stress

Asymmetric dimethyl arginine (ADMA) and oxidative stress may also contribute to endothelial dysfunction in Fontan patients. Asymmetric dimethyl arginine, which competitively inhibits eNOS and promotes endothelial cell apoptosis, is increased in Fontan patients [58,59]. Clinically, increased ADMA correlates with endothelial dysfunction in healthy and diseased populations [60], and reduced functional capacity in adult CHD patients [61].

Systemic oxidative stress in Fontan patients is characterized by raised serum methionine sulfoxide, which is produced when methionine reacts with reactive oxygen species (ROS) [58]. ADMA and angiotensin-II are known to enhance oxidative stress [58,62]. Superoxide rapidly reacts with circulating NO, directly reducing its bioavailability. This reaction yields peroxynitrite which causes eNOS uncoupling. Uncoupled eNOS is dysfunctional and produces superoxide instead of NO, further propagating endothelial dysfunction [63].

## 5 Significance of endothelial dysfunction in the SV-CHD population

### 5.1 Pulmonary vascular resistance and flow limitation

Pulmonary blood blow is abnormal in SV-CHD patients from birth and may further fall with initial palliations such as PA banding. Following the Glenn operation, pulmonary blood flow reduces drastically, and continues to be below normal after Fontan completion. This volume unloading results in pulmonary hypoplasia, leaving patients with small PA diameters and an elevated PVR [64,65]. Since endothelial dysfunction promotes vasoconstriction, it likely contributes to the PVR elevation in SV-CHD patients.

Pulmonary vascular resistance is a key determinant of SV-CHD function at each stage of palliation. In pre-Glenn patients, elevated PVR and PA pressures are associated with increased peri-operative mortality [66]. Following Fontan completion, those with higher pre-operative PVR have delayed recovery and required high-volume resuscitation [67]. In Fontan patients, elevated PVR is associated with a reduced cardiac index, exercise capacity, and elevated central venous pressure. It is also associated with worse long-term outcomes such as cirrhosis, protein-losing enteropathy, thromboembolism, heart failure hospitalization, atrial arrhythmias, and death [68].

Egbe et al. demonstrated that Fontan patients have an impaired pulmonary vascular reserve; they have an abnormal increase in PA pressures with increases in pulmonary blood flow (e.g. during exercise). The degree of pulmonary vascular reserve impairment correlated with endothelial dysfunction and indices of end-organ dysfunction including abnormal exercise capacity, renal function, and brain natriuretic peptide (BNP) [69].

Measurement of PVR in Fontan patients is often difficult and underestimated due to abnormal flow patterns. An increase in PVR and trans-pulmonary gradient is observed immediately after heart transplant, unmasking the true pulmonary state in these patients [70]. Raised pre-transplant PVR increases mortality in both pediatric and adult heart transplant recipients [71,72]. Due to its unique complexities, an elevated PVR may pose additional challenges in post-transplant Fontan patients, which are yet to be explored.

### 5.2 Exercise limitation

Exercise capacity is reduced in SV-CHD patients, and continues to decline after Fontan completion [[73], [74], [75]]. Reduced exercise capacity increases the risk of hospitalization and mortality in adult CHD and Fontan populations [6,76].

The mechanism of exercise limitation in pre-Fontan SV-CHD patients is not fully defined, but hypoxia, ventricular dysfunction, and an elevated PVR have been implicated [73,77]. Unlike healthy individuals, Fontan patients fail to increase pulmonary blood flow during exercise due to the absence of a sub-pulmonary ventricle, an elevated PVR, and reduced PVR responsiveness [78]. Endothelial function correlates with both pulmonary vascular reserve [69] and exercise capacity (VO_2max_) [40,79,80] in this population, suggesting a possible mechanistic link.

### 5.3 Thromboembolisms

SV-CHD patients have an increased risk of thrombotic complications including intravascular and intracardiac thrombosis, cerebrovascular disease, and pulmonary embolism. A combination of endothelial dysfunction, arrhythmia, flow limitation, and hypercoagulability is thought to underlie these events [81,82]. According to previous meta-analysis, aspirin and warfarin are equally effective for thromboprophylaxis in Fontan patients [83]. As such, it is unclear whether platelet hyperactivity or coagulation abnormalities are more dominantly involved. Endothelial dysfunction may be the key to a better understanding, as it promotes thrombosis through both these pathways [84]. Fontan patients with a history of thromboembolism have lower plasma levels of TM [32], and higher levels of vWF and P-Selectin [37], which possibly contribute to the hypercoagulable phenotype of this population.

### 5.4 Liver dysfunction

An important complication of SV-CHD is liver disease, but its point of onset and trajectory is not fully defined. Abnormal liver echotexture has been reported in 10 % of Glenn patients prior to Fontan completion [85]. Post-mortem studies also found evidence of liver fibrosis in a majority of those who died within 30 days after Fontan completion. Since liver fibrosis is considered a chronic process, its onset likely preceded Fontan completion [86].

Regardless, liver dysfunction is found almost universally in Fontan-palliated patients [87]. Late in the course of the Fontan circulation, this liver disease may progress to cirrhosis, and in some cases, portal hypertension [87]. Within the Fontan population, features of portal hypertension are associated with a need for transplantation, hepatocellular carcinoma, and death [88]. In general, intrahepatic sinusoidal endothelial dysfunction contributes to the progression of liver cirrhosis and portal hypertension [89]. Given the prolonged exposure to hemodynamic disturbances, SV-CHD patients may experience a greater degree of sinusoidal endothelial dysfunction, accelerating the progression of liver disease. However, the characteristics of this dysfunction and its role in Fontan-associated liver disease (FALD) are currently unknown and require further study.

### 5.5 Pregnancy

Normal pregnancy leads to dramatic cardiovascular changes including a hypercoagulable and pro-arrhythmogenic state, hypervolemia, autonomic changes, and reduced ventricular function. These changes pose significant challenges to the Fontan circulation [90]. Cardiovascular complications in pregnant Fontan patients can include acute (on chronic) heart failure, arrhythmias, thromboembolism, and rarely worsening of Fontan-associated liver and kidney dysfunction [90,91]. Post-partum hemorrhage also occurs more frequently [[90], [91], [92]], which may relate in part to the antiplatelets and anticoagulants used to mitigate the additional thrombotic risk in this group. Fetal and neonatal complications are markedly elevated, with a 45 % miscarriage rate reported. Of successful live births, more than half are born premature and one-fifth are small for gestational age. Several factors are thought to underlie these complications, including placental, uterine, hemodynamic, and neurohormonal abnormalities [92]. Failure of normal placentation, which depends on endothelial function and eNOS signaling, is a key risk factor in Fontan pregnancies [[93], [94], [95]]. However, a definitive link between endothelial dysfunction and abnormal placentation or poor pregnancy outcomes in the Fontan population is yet to be demonstrated.

The pathophysiology of pre-eclampsia involves abnormal placentation and release of several vasoactive factors resulting in widespread endothelial dysfunction with multi-organ involvement. Women with a history of pre-eclampsia continue to experience endothelial dysfunction even long after the pregnancy [96]. Hence, pregnant Fontan patients who experience pre-eclampsia may experience augmented levels of endothelial dysfunction.

## 6 Potential interventional strategies targeting endothelial dysfunction

### 6.1 Timing of fontan completion

Late Fontan completion (age >7 years) is associated with an increased risk of mortality and Fontan failure [45], whereas earlier completion (<3 years) results in a higher cardiac index and exercise capacity years after the procedure [97]. Furthermore, a negative correlation is seen between duration of cyanosis (SaO_2_ <80 %) before Fontan completion and brachial artery FMD [38]. Earlier Fontan completion reduces exposure to cyanosis, a potential causal mechanism, possibly improving long-term endothelial function.

### 6.2 Exercise habits and interventions

Exercise benefits Fontan patients by improving exercise capacity and quality of life [98], but its effects on pre-Fontan patients are not known. Early childhood is a crucial period for growth and development. In healthy pre-school aged children, simple physical activity interventions result in better motoric and cognitive development, building the foundation for a healthy and active lifestyle [99]. Interventions implemented in pre-Fontan patients could potentially enhance exercise participation in later years, though this has not yet been explored in research. In Fontan patients, early adoption of exercise behaviors leads to long-term improvements in hemodynamics, pulmonary function, hepatorenal function, and body composition [100].

Exercise improves endothelial function in heart failure, coronary artery disease, and hypertension, whereas a sedentary lifestyle and obesity are associated with poor endothelial function [7]. The skeletal muscle pump activated during lower limb exercise creates a pulsatile blood flow in the pulmonary circulation [101], the absence of which is thought to underlie pulmonary endothelial dysfunction in SV-CHD patients. Understanding how exercise influences endothelial function in these patients could facilitate more targeted exercise interventions.

### 6.3 Pharmacological interventions targeting endothelial dysfunction

#### 6.3.1 Pulmonary vasodilators and endothelin receptor antagonists

Whilst Fontan patients often do not fulfill typical criteria for pulmonary hypertension, they present with an elevated PVR which is critical to their overall function. Based on this, it has been suggested that pulmonary vasodilators might be beneficial [102]. However, a recent meta-analysis failed to show improvements in measures of exercise capacity and mortality with use of pulmonary vasodilators in Fontan-palliated patients [103]. The large-scale Fontan Udenafil Exercise Longitudinal (FUEL) randomized-controlled trial failed to show a significant improvement in peak oxygen consumption or endothelial function in adolescent Fontan patients treated with Udenafil. Nevertheless, the intervention group showed improvements in several exercise parameters at anaerobic threshold, including oxygen consumption, ventilatory equivalents of carbon dioxide (VE/VCO2), and work rate [104], indicating benefit at moderate levels of exercise.

Endothelin receptor antagonists (ERAs) directly modulate endothelial dysfunction [15]. *In vitro*, addition of Bosentan to endothelial cells cultured with ET-1 reverses the inhibition of eNOS expression and prevents further eNOS down-regulation, indicating its endothelium protective properties [105]. Clinically, Bosentan has been shown to improve endothelial function in several patient groups [[106], [107], [108], [109]].

The effect of ERAs on endothelial function in SV-CHD patients is unknown, but their effects on clinical outcomes have been studied. In a previous report, 8 pre-Fontan patients (3 pre-Glenn, 5 post-Glenn) were unable to undergo the Fontan procedure due to an elevated PVR and mean PA pressure. Both parameters reduced significantly following 6–12 months of Bosentan therapy, allowing all patients to undergo Fontan completion [110].

Similarly, ERAs reduce PVR in Fontan-palliated patients of all age groups [111]. The effects of ERAs on exercise capacity are contradictory and inconclusive (Table 1). While exercise capacity improved in a few studies[[111], [112], [113], [114], [115]], there were no effects in others [[116], [117], [118]]. A meta-analysis of three RCTs shows no overall effects of ERAs on exercise capacity [103]. This is corroborated by the recent RUBATO trial, which showed no benefit in exercise capacity after treatment with Macitentan [119]. Key limitations as with other Fontan studies are small sample sizes with significant phenotypic heterogeneity, a short intervention period, and high drop-out rates. It has been postulated that some sub-populations may be more responsive than others. Patients with an LT or extra-cardiac conduit type Fontan appeared to be more responsive to Bosentan compared to APC Fontan, but this was not backed by statistical evidence [113]. Besides this, better treatment effects have been reported in patients with a higher ventilatory efficiency at peak exercise [112].

**Table 1:** The use of endothelin receptor agonists in Fontan-Palliated patients.

Study	Study Design and Population	Baseline Characteristics of Patients receiving ERAs (Mean/Median)			Main Results
		Age (years)	Fontan duration (years)	Arterial Saturation (%)	
Ovaert et al. (2009) [116]	Open-label non-controlled trial 10 patients with a failing Fontan (30 % EC) circulation receiving Bosentan for 6 months. (9 completed the protocol)	12.1	7.8	86.0	No change in exercise capacity (6MWD, VO_2max_), oxygen saturations, or quality of life. Five responders identified, but no specific predictors reported.
Bowater et al. (2012) [117]	Open-label non-controlled trial 6 adult Fontan patients (17 % EC) (NYHA class ≥ II) receiving Bosentan for 6 months (all completed the protocol)	32.7	NA	92.3	No change in exercise capacity (6MWD, VO2_max_) NYHA class improved in 5/6 patients (p = 0.04). No predictors identified.
Schuuring et al. (2013) [118]	Open label RCT 42 adult Fontan patients (52 % EC) randomized to receive 6 months of Bosentan or 3 months of no treatment followed by 6 months of Bosentan. (32 completed the protocol)	28.0	NA	94.0	No effect of Bosentan on VO2_max_, NYHA class, cardiac output, or quality of life. No predictors of response identified
Hebert et al. (2014) [112]	Double-blind RCT 75 clinically stable Fontan patients (89 % EC) randomized to receive 14 weeks of Bosentan or Placebo. (69 patients completed the protocol).	20.3	NA	NA	Improved VO_2max_ compared to placebo (net treatment effect = 1.39 mL/Kg/min, p = 0.02). Higher odds of NYHA class improvement compared to control (p = 0.009). Treatment response better in patients with a higher Ve/VCO_2_ and Ve/VO_2_ at peak, and higher Ve/VCO_2_ at anaerobic threshold.
Derk et al. (2015) [113]	Open-label non-controlled trial 10 adult Fontan patients (40 % EC) receiving Bosentan for 4 months. (7 completed the protocol).	34.0	12.9	89.0	Improved 6MWD (p = 0.03) and MRI-derived cardiac output (p = 0.03). Better response was seen in subjects with a worse baseline NYHA class and LT/ECC vs AP type Fontan
Cedars et al. (2016) [114]	Double-blind cross-over RCT 19 adult Fontan patients (79 % EC) randomized to 12 weeks of Ambrisentan or placebo. Following a 2 week washout period, the groups were switched for 12 weeks.	24.9	19.7	90.6	Treatment resulted in improved VO_2max_ compared to baseline levels (p = 0.05).
Shang et al. (2016) [115]	Double-blind RCT 9 Fontan-palliated patients randomized to receive Bosentan or placebo for more than 1 year. All completed the protocol.	8.10	NA	NA	After 2 years, the Bosentan group had a higher 6MWD (p = 0.027) and a better NYHA functional class (p = 0.018).
Agnoletti et al. (2017)^a^ [111]	Prospective Cohort 24 Fontan-palliated patients (100 % EC) (8 children, 8 adolescents, and 8 adults).Patients received Bosentan (children and adolescents) or macitentan (adult) for 6 months. All completed the protocol. Double-blind RCT with open label extension	A: 9.0 B: 16.5 C: 25.5	A: 4.5 B: 11.5 C: 13.0	A: 96.5 B: 100.0 C: 96.0	PVR reduced in all groups (p < 0.01 in all groups) Indexed systemic output increased for adolescents (p = 0.04) and adults (p = 0.03) Indexed pulmonary output increased for children (p = 0.03) and adults (p = 0.002) VO_2max_ increased in adolescents (p = 0.02) but not in children and adults.
Clift et al. (2024) [119]	137 Fontan-palliated patients randomized to receive oral Macitentan or placebo for 1 year. Protocol was completed by 92.7 % of patients	23.2	18.3	92.8	No difference in change in VO_2_ from baseline, neither at peak exercise nor at ventilatory anaerobic threshold. No differences in subgroup analysis identified.

ERA = Endothelin receptor antagonists. Fontan Duration = Years since Fontan completion. EC = Extracardiac. 6MWD = 6-min walking distance. VO_2max_ = maximal oxygen consumption. NYHA = New York Heart Association. NA = Information not available. RCT = Randomized-controlled trial. Ve/VCO_2_ = Minute ventilation relative to CO_2_ production. Ve/VO_2_ = Minute ventilation relative to O_2_ consumption. ET-1 = Endothelin-1. PVR = Pulmonary vascular resistance.

a Patients in the study by Agnoletti et al. were stratified by age. A = Children. B = Adolescents. C = Adults.

#### 6.3.2 ACE-inhibitors

Since angiotensin-II is elevated and possibly contributes to endothelial dysfunction in Fontan patients, the renin-angiotensin-aldosterone system (RAAS) is a potential therapeutic target. In other diseases, ACE-inhibitors and angiotensin receptor blockers reportedly improve endothelial function [7]. However, both in pre- and post-Fontan patients, treatment with ACE-inhibitor has not been shown to influence endothelial function [17,[38], [39], [40]]. Fontan patients with RAAS-upregulating polymorphisms have worse diastolic dysfunction and higher BNP levels, both of which are associated with poor long-term outcomes [120]. ACE-inhibitors could perhaps benefit this subset of patients, but this is yet to be studied.

#### 6.3.3 Oxidative stress and vitamin C

An important cause of endothelial dysfunction is oxidative stress. Studies have attempted to elucidate the effects of naturally occurring antioxidants (e.g. vitamins C and E) on endothelial function, but with mixed results. A double-blind RCT found no effects of high dose Vitamin C on peripheral endothelial function or exercise capacity in Fontan patients. However, sub-group analysis found that in patients with severely reduced endothelial function (i.e. below the 25th percentile for healthy controls), vitamin C supplementation normalized endothelial function more often than placebo, indicating greater benefit in a subset of poorly functioning patients [121].

#### 6.3.4 The possible role of statins in Fontan-associated liver disease (FALD)

Recent evidence suggests that statins may benefit patients with liver cirrhosis, which is reviewed in depth elsewhere [122]. Statins reduce portal pressure, risk of decompensation, progression to hepatocellular carcinoma, and mortality in cirrhotic patients, at least partially due to improvements in sinusoidal endothelial dysfunction [89,122]. Liver disease is a significant source of morbidity in SV-CHD patients, and this may be an area for future study.

## 7 Future directions: finding the missing Jigsaw pieces

Current studies have built a strong foundation for the existence and significance of endothelial dysfunction in SV-CHD patients, and have provided a strong rationale for it as a potential therapeutic target. Nevertheless, several questions emerge from this review which remain unanswered. These are summarized in box 1 below.

Box 1 **Unanswered questions about endothelial dysfunction in the SV-CHD population.**

**Table:** 

**Evidence of endothelial dysfunction in the SV-CHD population**
1 What is the true onset of endothelial dysfunction in patients with SV-CHDs?
2 Is the endothelial expression of thrombomodulin constitutively reduced in SV-CHD patients?
3 Does the loss of normal variation in endothelial function persist at later ages in Fontan patients?
**Possible mechanisms of endothelial dysfunction in the SV-CHD population**
4 What is the relationship between arterial desaturation and endothelial dysfunction at each stage of SV-CHD palliation? Is hypoxia in early stages more relevant than later stages?
5 Does endothelial dysfunction correlate with sympathetic overactivation and increased neurohormonal activity in the SV-CHD population?
**Significance of endothelial dysfunction in the SV-CHD population**
6 Can endothelial dysfunction predict relevant long-term outcomes in the SV-CHD population?
7 How does an elevated pre-transplant PVR affect the post-transplant Fontan circulation?
8 What role does intrahepatic sinusoidal endothelial dysfunction play in the pathogenesis and progression of Fontan-associated liver disease (FALD)?
9 Does endothelial dysfunction correlate with worse pregnancy outcomes (e.g. increased risk of PPH or miscarriage) in women with the Fontan circulation?
10 Do Fontan patients with prior pregnancies complicated by pre-eclampsia have worse endothelial dysfunction than those without this history?
**Potential interventional strategies targeting endothelial dysfunction**
11 Is an earlier age of Fontan completion associated with better endothelial function in later life?
12 Can exercise interventions improve endothelial function in SV-CHD patients?
13 Are simple exercise interventions safe and beneficial in pre-Fontan populations?
14 What patient characteristics are associated with better responses to ERAs in SV-CHD patients?
15 Does ERA treatment improve endothelial function in the SV-CHD population?
16 Does improving endothelial function improve long-term outcomes in SV-CHD patients?
17 Does statin therapy improve sinusoidal endothelial dysfunction and Fontan-Associated Liver Disease?

Alt-text: Box 1

Endothelial dysfunction appears to be present at all stages of SV-CHD palliation, but its true onset remains unknown. Furthermore, current understanding of the underlying mechanisms remains speculative and are largely derived from other disease models. Endothelial dysfunction might influence the clinical trajectory of these patients in several domains, including exercise capacity, liver disease, thromboembolic risk, pregnancy, transplantation, and Fontan failure, but direct links are missing and must be sought. Collectively, answers to these questions would be able to guide interventional strategies in SV-CHD patients, which are still under development.

## 8 Conclusion

Endothelial dysfunction seems to play a central role in the pathophysiology of single ventricle congenital heart disease (SV-CHD), including patients who have undergone the Fontan procedure. The unique circulatory challenges in these populations—such as low cardiac output, altered venous return, and increased venous pressure—can lead to dysfunction of the endothelium, the inner lining of blood vessels. This dysfunction is often characterized by impaired vasodilation, increased vascular stiffness, and heightened inflammation, which may contribute to the development of various long-term complications, including heart failure, arrhythmias, and thromboembolic events.

Nevertheless, current data is limited and prospective studies which investigate the long-term clinical outcomes of endothelial dysfunction in this population are needed. These studies would elucidate the nature of how endothelial dysfunction progresses over time and its true impact on meaningful clinical outcomes. By systematically studying endothelial dysfunction in these populations, researchers can better understand the underlying mechanisms that drive poor outcomes in SV-CHD and Fontan patients. Unraveling this complex relationship would not only help identify key biomarkers that could be used to assess endothelial health but also guide the development of targeted therapies aimed at improving endothelial function. Ultimately, a deeper understanding of endothelial dysfunction in the context of single ventricle heart disease could significantly enhance clinical care for this increasingly recognized patient group. Timely interventions, supported by biomarkers and targeted therapies, could help improve quality of life, reduce the risk of complications, and potentially extend survival for this complex and growing population of patients.

## CRediT authorship contribution statement

**Raksheeth Agarwal:** Writing – review & editing, Writing – original draft, Resources, Methodology, Investigation, Conceptualization. **Louise E. Coats:** Writing – review & editing, Writing – original draft, Supervision, Conceptualization. **Ali N. Zaidi:** Writing – review & editing, Writing – original draft, Supervision, Conceptualization.

## Data statement

No data was generated for this review article.

## Funding sources

This research did not receive any specific grant from funding agencies in the public, commercial, or not-for-profit sectors.

## Declaration of competing interest

The authors declare that they have no known competing financial interests or personal relationships that could have appeared to influence the work reported in this paper; LEC is serving the IJCCHD Editorial Board but was not involved with the handling of this paper.
